# Impact of X-ray Exposure on the Proliferation and Differentiation of Human Pre-Adipocytes

**DOI:** 10.3390/ijms19092717

**Published:** 2018-09-11

**Authors:** Kateryna Shreder, Felicitas Rapp, Ioanna Tsoukala, Vanessa Rzeznik, Martin Wabitsch, Pamela Fischer-Posovszky, Claudia Fournier

**Affiliations:** 1 GSI Helmholtzzentrum für Schwerionenforschung, 64291 Darmstadt, Germany; k.shreder@gsi.de (K.S.); f.rapp@gsi.de (F.R.); i.tsoukala@gsi.de (I.T.); vanessa.rzeznik@web.de (V.R.); 2 Division of Pediatric Endocrinology and Diabetes, Department of Pediatrics and Adolescent Medicine, University Hospital Ulm, 89075 Ulm, Germany; Martin.Wabitsch@uniklinik-ulm.de (M.W.); Pamela.Fischer@uniklinik-ulm.de (P.F.-P.)

**Keywords:** adipocytes, adipokines, adipogenic differentiation, SGBS, radiotherapy, low dose radiation therapy, radiation response, photon irradiation

## Abstract

Radiotherapy is a widely used treatment option for cancer patients as well as for patients with musculoskeletal disorders. Adipocytes, the dominant cell type of adipose tissue, are known to constitute an active part of the tumor microenvironment. Moreover, adipocytes support inflammatory processes and cartilage degradation in chronic inflammatory diseases, i.e., rheumatoid and osteoarthritis. Since the production of inflammatory factors is linked to their differentiation stages, we set out to explore the radiation response of pre-adipocytes that may influence their inflammatory potential and differentiation capacity. This is the first study investigating the effects of X-ray irradiation on the proliferation and differentiation capacity of human primary pre-adipocytes, in comparison to Simpson–Golabi–Behmel Syndrome (SGBS) pre-adipocytes, an often-used in vitro model of human primary pre-adipocytes. Our results demonstrate a dose-dependent reduction of the proliferation capacity for both cell strains, whereas the potential for differentiation was mostly unaffected by irradiation. The expression of markers of adipogenic development, such as transcription factors (PPARγ, C/EBPα and C/EBPβ), as well as the release of adipokines (visfatin, adiponectin and leptin) were not significantly changed upon irradiation. However, after irradiation with high X-ray doses, an increased lipid accumulation was observed, which suggests a radiation-induced response of adipocytes related to inflammation. Our results indicate that pre-adipocytes are radio-resistant, and it remains to be elucidated whether this holds true for the overall inflammatory response of adipocytes upon irradiation.

## 1. Introduction

Growing evidence has been provided for an important role of adipose tissue as an active player in a variety of immunological and inflammatory processes [1,2]. Adipose tissue consists to a large extent of adipocytes. Other cell types of adipose tissue, called stroma vascular fraction (SVF), are stem cells, immune cells including lymphocytes and macrophages, fibroblasts, and endothelial cells [3]. The proportion between the different cell types within the adipose tissue is variable and depends both on the location of the adipose tissue deposit and the surrounding conditions [3,4,5]. Under inflammatory conditions, the fine regulation of the endocrine functions of adipose tissue leads to a chain reaction of pathological changes with respect to cell composition as well as expression and release of adipose tissue-specific factors [6,7,8]. The increased production of pro-inflammatory cytokines results in chronic low-grade inflammation and insulin resistance [9,10]. Both insulin resistance and dysregulation of the expression and release of key adipokines represent a core of systemic metabolic disorders and increase the risk of various serious diseases, including cancer and musculoskeletal diseases (MSD) [11,12].

Due to the presence of adipose tissue in different organs, adipocytes are located in close proximity to cancer cells in many solid tumors. The most prominent example is breast cancer, where adipocytes of the abundant fat tissue in the breast reside adjacent to tumor cells. Adipocytes secrete significant amounts of adipokines and other pro-inflammatory factors, which have been shown to promote tumor growth and expansion [13], and are associated with angiogenesis, which plays a critical role in tumor expansion [14,15,16]. In addition, interactions between adipocytes and cancer cells result in a synergistic increase of pro-inflammatory factors, such as IL-6 and CCL-2, which leads to the recruitment, infiltration and M2-polarization of macrophages, promoting an environment more permissive for tumor development [15,16]. Hence, adipocytes are part of the tumor microenvironment, orchestrating together with macrophages and the cancer cells several signaling pathways that are essential for the tumor development and progression.

Musculoskeletal diseases are usually associated with chronic inflammation, which is accompanied by reduced mobility and pain. Adipokines, which are primarily released by adipose tissue located next to joints, were found to be elevated in serum and synovial fluid of patients with MSD [17,18]. In rheumatoid arthritis (RA), adipokines contribute to the inflammation process in the joint by inducing the expression of pro-inflammatory cytokines such as IL-6, IL-8, and MMP-1 in synovial fibroblasts and other synovial cells [19,20,21]. This in turn leads to the activation of bone resorbing cells, i.e., osteoclasts, and finally to cartilage degradation and bone erosion.

Radiotherapy has long been established as an important treatment modality for cancer as well as for non-malignant diseases such as MSD. The main goal of tumor radiotherapy using high doses of radiation (single dose >1 Gy) is the induction of tumor cell death and activation of the immune system, whereas low dose radiation (single dose ≤1 Gy) is known to attenuate pre-existing inflammation and therefore is widely used for therapy of MSD [22,23]. In view of the important role of radiation therapy, studies on the radiation response of different tissues and cell types are emerging. However, data available about the radiation response of adipose tissue or adipocytes is scarce. So far, only two studies are published, which nonetheless provide controversial information. Despite comparable experimental conditions (5–10 Gy/137Cs, whole-body irradiation, mice), Poglio et al. reported the loss of adipose tissue after irradiation [24], while Jo et al. showed two times higher weights of adipose tissue in irradiated compared to unirradiated mice [25]. The differences reported by Poglio et al. and Jo et al. are probably related to the different time points, which were analyzed. Nevertheless, both authors agree on one point: ionizing radiation induces metabolic changes in adipose tissue.

Based on this, in the present study it was hypothesized that irradiation can induce metabolic changes in adipocytes, which could influence inflammatory and immune reactions in disease, as reported by others [16,20]. Considering the lack of knowledge about the impact of irradiation on pre-adipocytes and adipocytes, both residing in fat tissue close to tumors and inflamed joints, the radiation response of human primary pre-adipocytes with respect to release of adipokines was investigated in the present study. Since the ability of adipocytes to produce adipokines and pro-inflammatory factors is linked to their differentiation stages, the focus was set on the survival of irradiated pre-adipocytes and their differentiation capacity into mature adipocytes.

In addition, the radiation response of human primary (pre-)adipocytes was compared to that of SGBS cells. This cell strain isolated from the subcutaneous adipose tissue of an infant with a rare X-linked disorder, called Simpson–Golabi–Behmel syndrome (SGBS), exhibit a high capacity for adipocyte differentiation. Moreover, the morphology, gene expression pattern, and the biochemical function are very similar to that of human primary adipocytes [26,27,28]. SGBS cells, therefore, provide an appropriate model for studies on human adipocyte biology.

## 2. Results

### 2.1. X-ray Irradiation Inhibits Proliferation and Clonogenic Survival of Pre-Adipocytes

**Figure 1:**
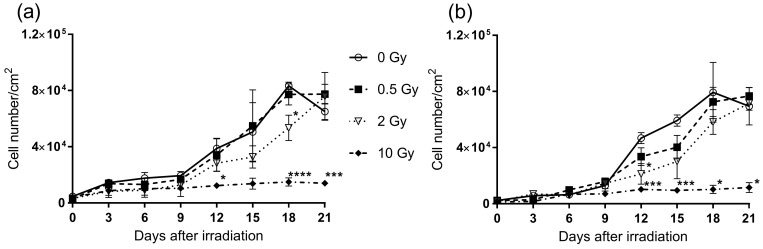
Proliferation of human pre-adipocytes after X-ray irradiation. (**a**) Human primary pre-adipocytes and (**b**) SGBS pre-adipocytes were exposed to 0.5, 2 and 10 Gy X-rays and cultivated over 21 days. Cell counting was performed on the indicated days. Mean, SEM, *N* = 3, * indicates significant differences from sham-irradiated samples on the same day; * *p* ≤ 0.05, *** *p* ≤ 0.001, **** *p* ≤ 0.0001, using a two-tailed *t*-test.

Radiation-induced changes in the proliferation capacity were monitored in primary and in SGBS pre-adipocytes over 21 days. The proliferation pattern of primary pre-adipocytes was very similar to that of SGBS pre-adipocytes (Figure 1a,b, respectively). In response to irradiation, the increase in cell numbers for both cell types was lower compared to sham-irradiated controls, which was visible from day 12 on. At the lowest dose (0.5 Gy), irradiation of pre-adipocytes caused no remarkable changes compared to controls, whereas SGBS cells were more sensitive, i.e., already showed lower cell numbers at 0.5 Gy compared to controls. Exposure to 2 Gy X-rays resulted in a strongly reduced proliferation capacity for both primary and SGBS cells, whereas proliferation of the cells exposed to 10 Gy was completely inhibited in both cell types.

**Figure 2:**
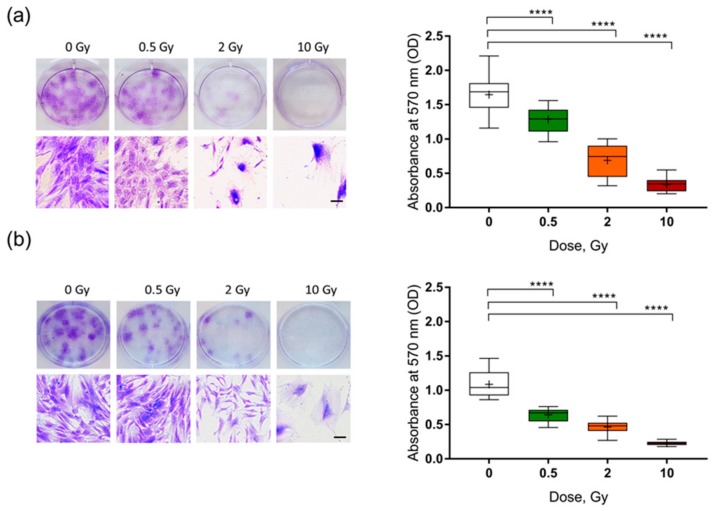
Clonogenic capacity of human pre-adipocytes after X-ray irradiation. (**a**) Human primary pre-adipocytes and (**b**) SGBS pre-adipocytes were exposed to 0.5, 2 and 10 Gy X-rays. Staining with crystal violet and quantitative analysis was performed on day 14. Boxplots show the median, Tukey whiskers (median ± 1.5 times interquartile range), and mean (+). *N* = 6, **** *p* ≤ 0.0001, using a two-tailed *t*-test. Scale bar represents 100 µm.

To determine the reproductive capacity of irradiated pre-adipocytes, a crystal violet cell proliferation assay was performed. Inspection by eye revealed that the formation of viable colonies in both primary (Figure 2a) and SGBS pre-adipocytes (Figure 2b) was inhibited in the irradiated compared to sham-irradiated cells. This was reflected by a significant, dose-dependent decrease in the absorbance of crystal violet at 570 nm.

### 2.2. Differentiation Potential of Pre-Adipocytes is Mostly Unaffected by X-ray Irradiation

**Figure 3:**
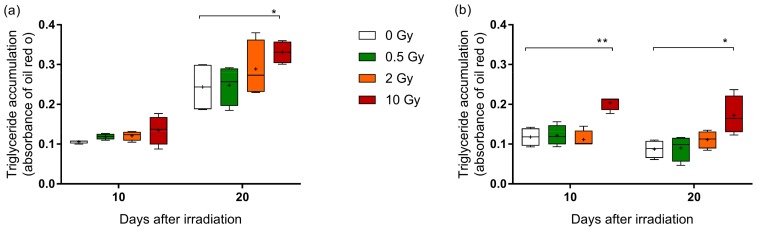
Effect of X-ray irradiation on triglyceride accumulation in human adipocytes. (**a**) Human primary pre-adipocytes and (**b**) SGBS pre-adipocytes were exposed to 0.5, 2, and 10 Gy X-rays and differentiation was initiated. The quantification of triglyceride accumulation was performed on day 10 and 20 after irradiation (medium change was not performed). Boxplots show the median, Tukey whiskers (median ± 1.5 times interquartile range) and mean (+). *N* = 4, * *p* ≤ 0.05, ** *p* ≤ 0.01, using a two-tailed *t*-test.

To evaluate whether ionizing radiation affects the differentiation process from pre-adipocytes to adipocytes, primary and SGBS pre-adipocytes were X-irradiated. The differentiation was initiated immediately after exposure and analyzed after 10 and 20 days by quantifying the amount of accumulated triglycerides in primary adipocytes (Figure 3a) and SGBS adipocytes (Figure 3b). In both cell types, only the exposure to 10 Gy of X-rays caused a significantly higher triglyceride amount when compared to sham-irradiated cells. Interestingly, the overall lipid accumulation in primary cells increased from day 10 to day 20, while no difference over time was observed in SGBS cells.

**Figure 4:**
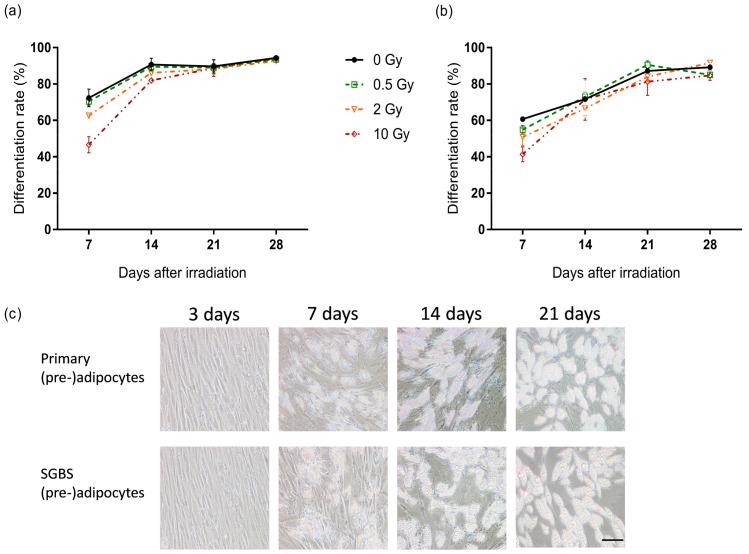
Effect of X-ray irradiation on adipogenic differentiation. (**a**) Human primary pre-adipocytes and (**b**) SGBS pre-adipocytes were exposed to 0.5, 2 and 10 Gy X-rays and differentiation was initiated. The cells were stained with Oil Red O at indicated days and the differentiation rate was calculated as a ratio of the number of differentiated cells and the total cell number. Mean, SEM, *N* = 3; (**c**) Representative pictures of (pre-) adipocytes at different stages of differentiation. Scale bar represents 100 µm.

To further characterize the effects of X-ray irradiation on the differentiation process of (pre-)adipocytes, the percentage of differentiated cells (differentiation rate) was evaluated over time in both cell types, starting on day 7, since intracellular lipid droplets are only visible from this stage on (Figure 4c). Representative Oil Red O stainings were given in Figure S1. As shown in Figure 4a,b, already on day 7 after onset of differentiation, sham-irradiated primary and SGBS cells reached a differentiation rate of 72% and 61% respectively. This increased to 90% (primary cells) and 87% (SGBS cells) by day 21. At this time point, the differentiation was accomplished; longer cultivation of the cells did not result in a further increase of differentiated cells. Radiation-induced changes of the differentiation process were not detectable, except a slight, dose-dependent delay of the differentiation rate at day 7 observed in both cell types. However, in both cases this effect became weaker in the course of further cultivation.

**Figure 5:**
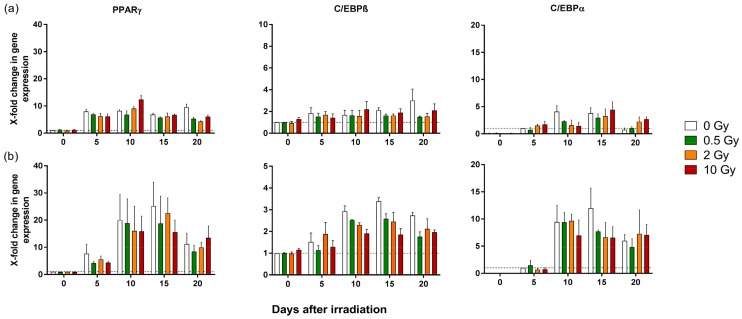
Gene expression of transcription factors in human adipocytes. (**a**) Human primary pre-adipocytes and (**b**) SGBS pre-adipocytes were irradiated with 0.5, 2, and 10 Gy X-rays and differentiation was initiated. Gene expression of PPARγ, C/EBPβ, and C/EBPα was analyzed on the indicated days using quantitative polymerase chain reaction qPCR. The data are normalized to GAPDH gene expression and to the gene expression on day 0 (for PPARγ and C/EBPβ) or on day 5 (for C/EBPα). The X-axis represents days after irradiation and the Y-axis is the relative fold change in gene expression. Mean, SEM. *N* = 3.

At the molecular level, adipogenesis is characterized by a tightly regulated interplay of stimulatory and inhibitory transcription factors. The critical factors for the complete and seamless adipocyte development are the Perixosome proliferator-activated receptor gamma (PPARγ) and the CCAAT/enhancer-binding proteins (C/EBPs), which are rapidly expressed after the induction of differentiation [29]. Therefore, as a next step, the impact of X-ray irradiation on the expression of these major adipogenic transcription factors (PPARγ, C/EBPβ and C/EBPα) were investigated. After irradiation and initiation of differentiation, RNA from both primary and SGBS cells were isolated at the indicated time points. Although no significant differences in the expression of PPARγ, C/EBPβ, and C/EBPα between irradiated and sham-irradiated cells were found, the analysis revealed a trend towards a reduced expression level of these genes (Figure 5). Notably, primary (pre-)adipocytes (Figure 5a) showed much lower expression levels of adipogenic transcription factors during differentiation compared to SGBS cells (Figure 5b), although the temporal pattern of gene expression was found to be very similar.

### 2.3. X-ray Irradiation Does Not Significantly Affect Adipokine Release by Human Adipocytes

**Figure 6:**
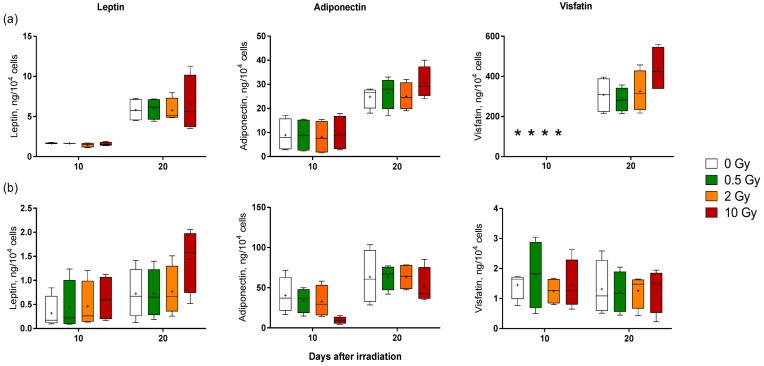
Effect of X-ray irradiation on the adipokine release by human adipocytes. (**a**) Human primary pre-adipocytes and (**b**) SGBS pre-adipocytes were exposed to 0.5, 2 and 10 Gy X-rays and differentiation was initiated. Cell supernatants were collected on day 10 and 20 after irradiation and the amount of adipokines leptin, adiponectin, and visfatin were measured using enzyme-linked immunosorbent assay ELISA. Boxplots show the median, Tukey whiskers (median ± 1.5 times interquartile range) and mean (+). X-axis represents days after irradiation and Y-axis is the amount of adipokines released. *N* = 4, * value below detection limit.

To assess the possible radiation-induced changes in the release of adipokines, the levels of selected adipokines were measured in cell supernatants of primary adipocytes (Figure 6a) and SGBS adipocytes (Figure 6b) 10 and 20 days after the irradiation and differentiation start (medium change was not performed). For primary adipocytes, an upregulation of the release of leptin and adiponectin was observed between 10 and 20 days, but irrespective of irradiation. With respect to the release of visfatin, the measured amount was under the detection limit 10 days after the start of differentiation and had dramatically increased by day 20. Moreover, cells exposed to 10 Gy X-rays exhibited a higher, even though not statistically significant, visfatin concentration than sham-irradiated cells (Figure 6a). Under the culture conditions chosen, a different adipokine release pattern was observed in SGBS adipocytes. Although the amount of released leptin and adiponectin was higher on day 20 compared to day 10, the differences were not as clear as in primary adipocytes. Moreover, compared to sham-irradiated cells, the exposure to 10 Gy X-rays caused a slight increase in the leptin level in SGBS cells, while the adiponectin level was reduced. In contrast to primary adipocytes, SGBS cells had already released visfatin on day 10 after differentiation start, but in hardly detectable amounts, which did not increase between day 10 and 20. In addition, the visfatin release by SGBS adipocytes did not show any recognizable radiation-induced changes.

Taken together, our results show that the human primary subcutaneous pre-adipocytes as well as SGBS (pre-) adipocytes were resistant to X-ray radiation with respect to typical radiation responses. In addition, the findings of this study demonstrate that primary and SGBS cells displayed a similar radiation response.

## 3. Discussion

In the study presented here, the radiation response of human primary pre-adipocytes with respect to differentiation and release of adipokines was investigated. Adipocytes produce several factors, which influence different biological processes, including inflammatory and immune responses and have many implications for human diseases [10,30]. Since the expression and release of adipogenic factors is linked to the differentiation stage, a thorough understanding of the differentiation process could contribute important understanding to the role of adipocytes in certain diseases such as chronic inflammatory and cancer diseases.

It has been reported that the presence of adipokines can modify the behavior of cells located in close proximity to fat tissue [31], and it has been assumed that this is caused, among other things, by changes in adipokine profile. Furthermore, adipokines, which are primarily released by adipocytes, are established differentiation markers of adipocytes and known to be involved in inflammation and the immune response [8,17,19]. Several studies demonstrated that an imbalanced profile of adipokine expression and release, including elevated levels of leptin, visfatin, and reduced adiponectin supports tumor growth and invasion [6,16,32]. Also, in chronic inflammatory diseases such as musculoskeletal diseases (MSD), an imbalanced adipokine profile was reported to induce the expression and release of pro-inflammatory factors in other cell types, contributing to establishment of inflammation and disease progression [17,20].

An assessment of the link between adipocyte differentiation and release of adipokines could also highlight a putative participation of adipocytes in the mechanisms elicited during treatment of these diseases, for example in radiotherapy, which is widely used for treatment of both diseases. It is established that low dose radiotherapy with photons and radon exposure inhibits chronic inflammation in degenerative bone and other diseases [33,34,35], whereas high-dose radiotherapy of tumors triggers inflammation [22,36]. At the onset of the study presented here, it was hypothesized that irradiation can induce metabolic changes in adipocytes and/or changes in the differentiation of pre-adipocytes into adipocytes. This could appear as changes in proliferation, lipid accumulation, and adipokine release.

In previously reported studies, irradiated mesenchymal stem cells (MSC) turned out to be radio-resistant with respect to their differentiation capacity into the respective lineages, i.e., adipocytes, osteoblasts, and chondrocytes [37,38,39,40,41]. However, no data is available regarding the radiation effects elicited in irradiated pre-adipocytes and their differentiation to mature adipocytes, both the most abundant cell types in fat tissue [7]. The goal of the present study was to fill this gap. Therefore, relevant changes in irradiated primary pre-adipocytes, of human subcutaneous origin, were assessed and compared to those detected in SGBS (pre-)adipocytes, which constitute an often used in vitro model for human adipocytes [26].

The first question raised in the present study was to what extent pre-adipocytes survive radiation exposure. Our results revealed that the proliferation rate and clonogenic capacity of both primary and SGBS pre-adipocytes were decreased; for clonogenic capacity this was already detectable at a low dose (0.5 Gy). Thus, the regenerative potential of irradiated pre-adipocytes was reduced. Furthermore, the weak apoptotic response of pre-adipocytes after irradiation (Figure S2) indicates that the observed reduced proliferation rate was likely a result of cell cycle arrest rather than cell death. As pre-adipocytes arise from MSC, it was not surprising that their proliferation pattern after irradiation was very similar to that of mesenchymal stem cells [38,42,43] and other cells of mesenchymal origin [44], which show primarily cell cycle arrest and senescence, and not apoptosis.

A link to the regenerative potential of pre-adipocytes is their ability to differentiate into mature adipocytes, a key feature of pre-adipocytes. The most prominent adipogenic feature is the ability to accumulate lipids. The results of our study revealed that adipocytes tended to increase lipid accumulation after irradiation, in both primary and SGBS cells compared to sham-irradiated cells and most pronounced and significantly elevated at the highest dose (10 Gy), occurring at a late stage of differentiation. Notably, higher lipid accumulation compared to controls occurs, although the rate of differentiated cells was comparable. However, in contrast to the detected increase in lipid accumulation after irradiation, the differentiation rate did not differ between irradiated and sham-irradiated cells. This is in line with more complex observations in the organism, i.e., observation of obesity as a late effect after radiotherapy [45,46], and experimental in vivo results obtained by Jo et al. [25], who also reported an increased size of the irradiated adipocytes compared to controls.

Taken together, this suggests a radiation-induced lipid accumulation after relatively high doses, leading to an increase in cell size (hypertrophy) rather than in cell number (hyperplasia) of adipocytes. Since it was reported that the hypertrophied adipocytes recruit activated macrophages and release a variety of factors, which promote inflammation and insulin resistance [12,47], it can be speculated that exposure of adipose tissue to high doses of radiation can be an additional risk factor for the development of inflammation caused by a radiation induced metabolic shift and a key reason for radiotherapy-associated obesity in cancer survivors [46]. However, as the single dose administered to the patient rarely exceeds the highest dose used in the study (10 Gy), but the overall dose in a fractionation regimen exceeds this dose, and this idea needs further investigation.

The differentiation process of adipocytes is subjected to a close regulation and control by a variety of growth- and transcription factors. PPARγ appears prior to the activation of many other adipogenic genes and is, together with C/EBPα, essential not only for induction of adipogenesis, but also for a growth arrest, which is required for adipocyte maturation [48]. Along with C/EBPβ, these genes determine the differentiation process of adipocytes. In our study, the gene expression of PPARγ, C/EBPα, and C/EBPβ in both primary and SGBS cells was mostly unaffected by irradiation, suggesting that human pre-adipocytes and mature adipocytes did not change their established molecular profile upon X-ray irradiation. These results are in agreement with the unaffected differentiation rate found in the same cells. Interestingly, the above-mentioned increase in lipid accumulation is not reflected by the expression of PPARγ, C/EBPα, and C/EBPβ, and suggests a modulation of other factors after irradiation. In contrast to the investigation of pre-adipocytes in our study, Li et al. and Nicolay et al. [43,49] investigated the differentiation capacity of MSC after irradiation in a dose range comparable to our study. Li et al. found that the differentiation process of adipocytes after irradiation with low doses of photons was not significantly altered, but for higher doses, a reduced number of mature adipocytes was observed [43]. Similar to the data presented here, Nicolay et al. showed that expression of various adipogenic or osteogenic markers in irradiated MSC remained unchanged, suggesting that at the transcriptional level, the differentiation was not affected [49].

Remarkably, in the first part of the study presented here, proliferation and cell differentiation patterns in SGBS as compared to primary (pre-) adipocytes were found to be similar, whereas the level of gene expression of transcription factors was much higher in SGBS cells compared to primary cells. Different gene expression profiles in primary and SGBS (pre-)adipocytes, both isolated from white adipose tissue (WAT), were already described by Yeo et al. [50]. According to their study, SGBS-cells can shift towards a brown adipocyte phenotype, which might reflect differences in function and composition of WAT in childhood and adulthood, because SGSB cells are derived from the WAT of a 3-month-old infant, whereas primary pre-adipocytes used in this and most other studies are derived from a subcutaneous adipose tissue of adults [50].

The next step was to assess whether the release of adipokines is changed in adipocytes after radiation exposure, thereby potentially contributing to the previously reported impact of radiation exposure on inflammation and immune response [35,51,52]. This is suggested by the significant influence of adipokines on the pathogenesis of the diseases which are treated with irradiation. For example, the results of our previous study revealed a significant decrease in visfatin serum levels of patients with MSD after low-dose radon therapy [35].

In the study presented here, it turned out that the adipokine release by adipocytes was increased during differentiation, but irradiation did not significantly change the adipokine release, irrespective of the doses administered. However, a trend to an enhanced release after a high-dose exposure was observed at least for primary adipocytes. This suggests that radiation-induced changes in the release of the tested adipokines were not part of the direct radiation response of adipocytes.

Taken together, in the present study, the radiation response of human primary pre-adipocytes and SGBS (pre-)adipocytes were characterized for the first time. Radiation sensitivity turned out to be similar for both cell types. While proliferation capacity and clonogenic survival of pre-adipocytes were dose-dependently affected, no impact on the differentiation process of pre-adipocytes into mature adipocytes was observed. The same accounts for adipokine release, except for a trend to an increase at high doses. However, increased lipid accumulation, as observed after high-dose exposure, which is a hallmark of hypertrophy, may be part of the inflammatory response observed during or after the radiotherapy of tumors.

## 4. Materials and Methods

### 4.1. Cell Culture

Primary human subcutaneous pre-adipocytes (in the following referred to as the primary pre-adipocytes) were purchased from Lonza Ltd. The cells were cultured in pre-adipocyte growth medium (Lonza Ltd., Basel, Switzerland) until reaching confluence. For the differentiation into adipocytes, appropriate supplements (Lonza Ltd., Basel, Switzerland) including insulin, dexamethasone, 3-isobutyl-1-methylxanthin (IBMX), and indomethacin were added. Simpson–Golabi–Behmel syndrome (SGBS) pre-adipocytes were cultured and differentiated as previously described with rosiglitazone as a PPARγ agonist [26]. Due to their high capacity for adipogenic differentiation, SGBS cells are an excellent tool for studies of adipocytes in vitro [28]. In order to perform experiments with undifferentiated pre-adipocytes (either primary pre-adipocytes or SGBS), growth medium containing 10% FCS without differentiation factors was used for cell cultivation. Incubator conditions for both cell types were set to a relative humidity of 95%, 5% CO_2_ and 37 °C. Unless indicated otherwise, the medium change was performed two or three times a week.

### 4.2. Irradiation Procedure

Cells were exposed to single doses (0.5, 2 and 10 Gy) of X-ray irradiation (1 Gy/min). Irradiation was performed at room temperature using an X-ray tube (IV320-13, Seifert, Ahrensburg, Germany) with a tube voltage of 250 kV and a cathode current of 16 mA. A PTW-SN4 detector (PTW, Freiburg, Germany) was used for dosimetry. Controls were sham-irradiated cells.

### 4.3. Crystal Violet Cell Proliferation Assay

Primary pre-adipocytes and SGBS pre-adipocytes were plated at a density of 1 × 10^3^ cells in six-well plates and allowed to attach for 24 h before irradiation. After irradiation, cells were cultivated for 14 days, and the medium was changed once a week. Colonies were fixed with 3.7% formaldehyde and stained with 5% crystal violet (Sigma-Aldrich, St. Louis, MO, USA) in 10% methanol. For quantitative analysis, the stained cells were dissolved in 100% methanol and subjected to an absorbance measurement at 570 nm using a microplate spectrometer ELx808 (BioTek, Winooski, VT, USA.).

### 4.4. Analysis of Adipocyte Differentiation

Quantification of triglyceride accumulation: Lipid accumulation was quantified by the measurement of extracted Oil Red O (Sigma-Aldrich, St. Louis, MO, USA). Briefly, cells were washed with phosphate-buffered saline (PBS) (Merck, Kenilworth, NJ, USA) and fixed with 3.7% formaldehyde. After removing the formaldehyde and washing with distilled water, 60% isopropanol was added. Then cells were stained with Oil Red O. For the quantification of triglyceride accumulation, cells were carefully washed with distilled water and the extraction of Oil Red O was performed by adding 4% Triton X-100 (Sigma-Aldrich, St. Louis, MO, USA) in isopropanol, followed the measurement of absorbance at 540 nm.

Counterstaining of adipocyte nuclei: For the visualization of adipocyte nuclei, 4′,6-diamidino-2-phenylindole (DAPI) staining was performed. Cells were washed with distilled water after performing Oil Red O staining, and the DAPI staining solution (1 µg/mL, SERVA Electrophoresis, Heidelberg, Germany) was added. Afterwards, cells were washed twice with PBS and the slides were covered with Vectashield™ and a glass cover slip. Blue-stained nuclei were detected at a wavelength of 340 nm.

Determination of adipocyte differentiation rate: Cells were plated at a density of 1 × 10^4^ on chamber slides and cultivated until reaching confluence. Then, cells were irradiated and differentiation was initiated. Cells were stained with Oil Red O and DAPI on defined days and 14 randomly selected areas per slide were visually examined using an Olympus BX61 microscope (Olympus Corp., Tokyo, Japan). For this, all DAPI-stained cells and all Oil Red O-positive cells were counted and the differentiation rate (DR) was calculated as follows:

$$DR\;=\frac{n\;\left(Oil\;Red\;O\right)}{n\;\left(DAPI\right)}\times 100\%$$

### 4.5. Quantification of Adipokine Release in Cell Culture Supernatants

The concentrations of adipokines in cell culture supernatants were determined using an enzyme-linked immunosorbent assay (ELISA) and normalized to the cell number. Cell culture supernatants were collected 10 and 20 days after irradiation and initiation of differentiation and frozen at −80 °C prior to analysis. A medium change was not performed during the cultivation. ELISA for adiponectin and leptin (TECOmedical Group, Sissach, Switzerland) as well as for visfatin (AdipoGen Life Sciences Inc., Liestal, Switzerland) were carried out according to the manufacturer’s instructions.

### 4.6. Gene Expression Analysis During Differentiation

Cells were starved in a serum-free medium 24 h prior to RNA extraction. Total RNA was isolated from pre- or mature adipocytes on defined days using an RNeasy Mini Kit (Qiagen, Hilden, Germany) and reverse-transcribed to the complementary DNA (cDNA) using RevertAid™ First Strand cDNA Synthesis Kit (Thermo Fisher Scientific, Waltham, MA, USA) according to the manufacturer’s instructions. The real-time qPCR runs were performed using a StepOnePlus real-time PCR System (Applied Biosystems, Foster City, CA, USA). QuantiTect SYBR Green PCR Kit (Qiagen, Hilden, Germany), 100 ng cDNA/reaction, and Qiagen QuantiTect Primer assays (Qiagen, Hilden, Germany) were used for all experiments. Relative gene expression levels were calculated using the 2^−ΔΔ*C*t^ method normalized to the expression levels of a housekeeping gene GAPDH, and fold changes in expression were calculated relative to those of sham-irradiated samples. The specificity of PCR products was verified using a melting curve analysis. Amplification reactions for each sample were performed in duplicate. A non-template control was included in each real-time qPCR run.

### 4.7. Statistical Analysis

Statistical analysis was performed using a two-tailed *t*-test after checking for the normal distribution of the data points with a D’Agostino and Pearson test (Graph Pad Prism 6, Graph Pad Software, La Jolla, CA, USA). Probability values *p* < 0.05 were considered statistically significant.

## 5. Conclusions

The data presented here show that irradiation of pre-adipocytes decreased the number of proliferating pre-adipocytes, but did not significantly alter the differentiation process, including the release of the inflammation-related adipokines tested in this study. This suggests that pro- or anti-inflammatory effects, potentially exerted by adipose tissue upon radiation treatment and discussed as a trigger for modified inflammation, were caused by other cell types of the adipose tissue. These cells could either act directly following radiation exposure or indirectly via influence on adipocytes. However, it remains to be elucidated whether the observed radio-resistance of pre-adipocytes holds true for the overall inflammatory response of (pre-)adipocytes.

## Supplementary Materials

Supplementary materials can be found at http://www.mdpi.com/1422-0067/19/9/2717/s1.

## Abbreviations

C/EBP	CCAAT/enhancer-binding proteins
CCL-2	CC-chemokine ligand 2
cDNA	complementary DNA
CFU	colony-forming unit
DAPI	4′,6-Diamidin-2-phenylindol
ELISA	Enzyme-linked immunosorbent assay
IBMX	3-Isobutyl-1-methylxanthin
IL	Interleukin
MMP-1	Matrix metalloproteinase-1
MSC	mesenchymal stem cells
MSD	musculoskeletal diseases
PPARγ	Perixosome proliferator-activated receptor gamma
qPCR	quantitative PCR
RA	rheumatoid arthritis
SGBS	Simpson–Golabi–Behmel Syndrome
WAT	white adipose tissue
