# Supplementary material for: Impact of X-ray Exposure on the Proliferation and Differentiation of Human Pre-Adipocytes
Source: Int J Mol Sci. 2018 Sep 11;19(9):2717. doi: 10.3390/ijms19092717 (PMC6163807; doi:10.3390/ijms19092717)
Supplement: Supplementary file 1 [file ijms-19-02717-s001.zip › ijms-346724-author proof-supp-done.pdf]

International Journal of
Molecular Sciences

MDPI

Supplementary Material

Impact of X-ray Exposure on the Proliferation and Differentiation of Human Pre-Adipocytes

Kateryna Shreder [1], Felicitas Rapp [1], Ioanna Tsoukala [1], Vanessa Rzeznik [1], Martin Wabitsch [2], Pamela Fischer-Posovszky [2] and Claudia Fournier [1*]

[1] GSI Helmholtzzentrum für Schwerionenforschung, Department of Biophysics, 64291 Darmstadt, Germany; k.shreder@gsi.de, f.rapp@gsi.de, vanessa.rzeznik@web.de, i.tsoukala@gsi.de, c.fournier@gsi.de
[2] University Hospital Ulm, Department of Pediatrics and Adolescent Medicine, Division of Pediatric Endocrinology and Diabetes, 89075 Ulm, Germany; Martin.Wabitsch@uniklinik-ulm.de; Pamela.Fischer@uniklinik-ulm.de,
[*] Correspondence: c.fournier@gsi.de; Tel.: +49-6159-71-2692

Quantification of Adipogenic Differentiation by Oil Red O staining

In order to detect the differentiation stage, human primary pre-adipocytes and Simpson- Golabi-Behmel syndrome (SGBS) pre-adipocytes were cultivated according to chapter 4.1 and 4.4. Representative stainings with Oil Red O are shown in Figure S1 for 3 and 14 days to illustrate triglyceride accumulation.

X-ray Irradiation Does Not Increase Cell Death in Human Pre-Adipocytes

Mesenchymal stem cells do not show a pronounced apoptotic response upon ionizing irradiation [1]. For the present work, it was assumed that this accounts also for (pre-)adipocytes. To test this, the occurrence of cell death after irradiation was investigated in primary and SGBS cells.

Cell death in pre-adipocytes was evaluated 6, 24 and 48h after irradiation using flow cytometry following annexin V/ propidium iodide (PI) staining. Briefly, $1x10^6$ enzymatically harvested cells were washed with PBS, centrifuged for 5 min at 200× *g*, resuspended in 100 µl Annexin V/PI labeling solution (Roche, Mannheim, Germany) and incubated for 15 min at room temperature. Then, samples were diluted with 400 µl of incubation buffer and immediately analyzed using BD FACS Canto II machine (BD Biosciences). Data analysis was performed with FlowJo software (Ver. 7, BD Biosciences).

As shown in Figure S2, the death rate in sham-irradiated cells was approximately 2–3% at all time points, irrespective of the cell type. With respect to irradiation, for primary pre-adipocytes compared to sham-irradiated cells, no changes in the percentage of dead cells were observed (Figure S2b). For SGBS pre-adipocytes, the death rate was slightly increased in irradiated cells (2 and 10 Gy of X-rays) (Figure S2c). However, compared to the hyperthermia-induced death rate in SGBS-cells (67.7%, data not shown), the difference of about 3% between sham-irradiated and 10 Gy-irradiated SGBS-cells was not considered as an increase.

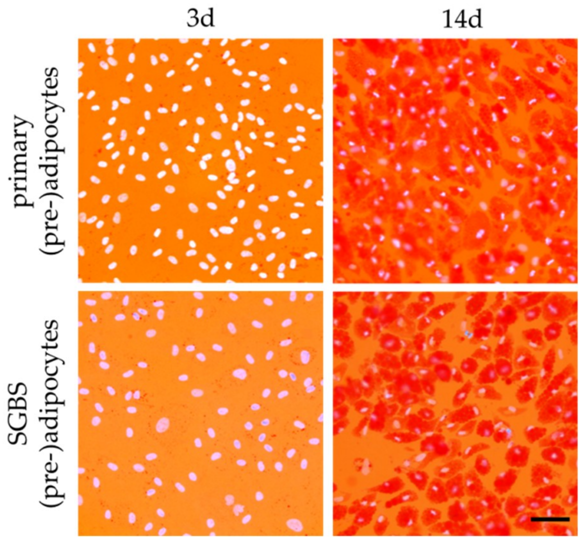

Figure S1. Adipogenic differentiation at different stages. Human primary pre-adipocytes and SGBS pre-adipocytes were stained with Oil Red O (red) and DAPI* (blue/white). To evaluate differentiation, the ratio of Oil Red O-positive cells to the total number of cells was calculated. Scale bar represents 100 µm. * DAPI- 4',6-diamidino-2-phenylindole

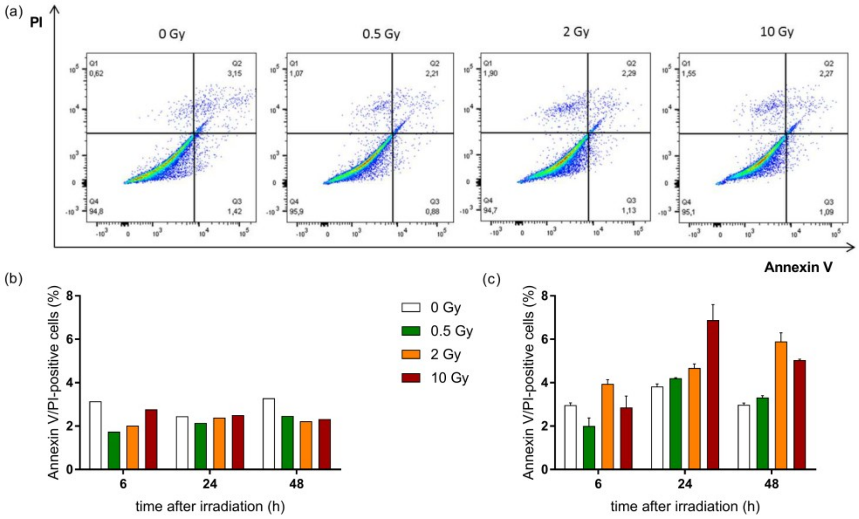

Figure S2. Effect of X-ray irradiation on cell death induction of human pre-adipocytes. Human primary pre-adipocytes and SGBS pre-adipocytes were exposed to 0.5, 2 and 10 Gy X-rays. For cell death evaluation, cells were stained with a combination of annexin V and propidium iodide (PI) and analyzed using flow cytometry. (***a***) Representative flow cytometry dot plots for analysis of apoptosis in primary pre-adipocytes 48h after irradiation. (***b***,***c***) Percentage of dead cells (Annexin V+/PI+) in primary pre-adipocytes or SGBS pre-adipocytes, respectively. *N* = 1 for primary cells, *N* = 2, Mean ± SD* for SGBS-cells. * SD – standard deviation

References

[1] N.H. Nicolay, R.L. Perez, R. Saffrich, P.E. Huber, Radio-resistant mesenchymal stem cells: mechanisms of resistance and potential implications for the clinic, Oncotarget. 6 (2015) 19366–19380.
